# Genome Features and *In Vitro* Activity against Influenza A and SARS-CoV-2 Viruses of Six Probiotic Strains

**DOI:** 10.1155/2021/6662027

**Published:** 2021-06-18

**Authors:** Irina V. Soloveva, Tatyana N. Ilyicheva, Vasiliy Yu. Marchenko, Oleg V. Pyankov, Anna G. Tochilina, Irina V. Belova, Vladimir A. Zhirnov, Nikolay I. Bormotov, Maksim O. Skarnovich, Aleksander G. Durymanov, Svetlana B. Molodtsova, Ekaterina I. Filippova, Alena S. Ovchinnikova, Anastasia V. Magerramova, Alexander B. Ryzhikov, Rinat A. Maksyutov

**Affiliations:** ^1^Academician I.N. Blokhina Nizhny Novgorod Scientific Research Institute of Epidemiology and Microbiology, 71, M. Yamskaya Street, Nizhny Novgorod 603950, Russia; ^2^Vector State Research Centre of Virology and Biotechnology, Koltsovo, Novosibirsk Region 630559, Russia; ^3^Novosibirsk State University, 2, Pirogov Street, Novosibirsk 630090, Russia

## Abstract

**Purpose:**

The aim of this work was to analyze the complete genome of probiotic bacteria *Lactobacillus plantarum* 8 RA 3, *Lactobacillus fermentum* 90 TC-4, *Lactobacillus fermentum* 39, *Bifidobacterium bifidum* 791, *Bifidobacterium bifidum* 1, and *Bifidobacterium longum* 379 and to test their activity against influenza A and SARS-CoV-2 viruses.

**Methods:**

To confirm the taxonomic affiliation of the bacterial strains, MALDI TOF mass spectrometry and biochemical test systems were used. Whole genome sequencing was performed on the Illumina Inc. MiSeq platform. To determine the antiviral activity, A/Lipetsk/1V/2018 (H1N1 pdm09) (EPI_ISL_332798) and A/common gull/Saratov/1676/2018 (H5N6) (EPI_ISL_336925) influenza viruses and SARS-CoV-2 virus strain Australia/VIC01/2020 (GenBank: MT007544.1) were used.

**Results:**

All studied probiotic bacteria are nonpathogenic for humans and do not contain the determinants of transmission-type antibiotic resistance and integrated plasmids. Resistance to antibiotics of different classes is explained by the presence of molecular efflux pumps of the MatE and MFS families. Cultures of *L. fermentum* 90 TC 4, *L*. *plantarum* 8 RA 3, and *B. bifidum* 791 showed a pronounced activity against influenza A viruses in MDCK cells. Activity against the SARS-CoV-2 virus was demonstrated only by the *L. fermentum* 90 TC 4 strain in VERO cells.

**Conclusions:**

The studied probiotic bacteria are safe, have antiviral activity, and are of great importance for the prevention of diseases caused by respiratory viruses that can also infect the human intestine.

## 1. Introduction

Probiotic bacteria have a number of unique properties and provide invaluable benefits to human health [1–3]. Lacto- and bifidobacteria in the body carry out important functions. They participate in metabolism, produce biologically active compounds (vitamins, neuropeptides, bacteriocins, etc.), play a role in immunogenesis, and provide colonization resistance, and in addition, they exhibit antiviral activity against some viral pathogens in vitro and in vivo [4–7]. The main mechanisms of the inhibitory action of probiotic bacteria on viruses are a general immunostimulating effect of probiotic strains, physical adsorption of viral particles on the surface of bacteria where exopolysaccharides play an important role, specific interaction of probiotic bacteria with viral particles, and production of active metabolites [7, 8].

It is known that human respiratory viruses replicate mainly in the respiratory tract, and therefore, it is believed that probiotics cannot influence the dynamics of the disease. However, it has been described that during an influenza pandemic and sometimes during seasonal epidemics, gastrointestinal diseases caused by the influenza virus are observed, especially in children. In addition, when humans are infected with highly pathogenic avian influenza viruses [9] or the novel SARS-CoV-2 coronavirus [10], the pathogen also infects the intestines of the host. In this regard, the relevance of studying the antiviral activity of probiotic strains of lacto- and bifidobacteria against respiratory viruses is beyond doubt.

The studied bacteria of the genera *Lactobacillus* and *Bifidobacterium* are used in Russia as probiotic producer strains. Their biological properties were investigated earlier using traditional microbiological and molecular genetic methods; however, whole genome sequencing of all strains has not been performed [11]. Analysis of the genomic sequences of the strains can confirm the safety of *L. plantarum* 8 RA 3, *L. fermentum* 90 TC 4, *L. fermentum* 39, *B. bifidum* 1, *B. bifidum* 791, and *B. longum* 379 bacteria for use as producers of probiotics and will also help to identify the determinants of antiviral activity.

The aim of this study was to analyze the genome-wide sequences of *L. plantarum* 8 RA 3, *L. fermentum* 90 TC 4, *L. fermentum* 39, *B. bifidum* 1, *B. bifidum* 791, and *B. longum* 379 probiotic bacteria and to study their antiviral activity against the A(H1N1 pdm09) influenza virus, the highly pathogenic influenza A(H5N6) virus, and the SARS-CoV-2 virus that caused the COVID-19 pandemic.

## 2. Materials and Methods

### 2.1. Objectives

We studied bacteria used in Russia for the production of medicinal probiotics: *Lactobacillus plantarum* 8 RA 3 (State Collection of Pathogenic Microorganisms and Cell Cultures SCPM-Obolensk No. 900811), *Lactobacillus fermentum* 90 TC-4 (SCPM-Obolensk No. 900812), *Lactobacillus fermentum* 39 (SCPM-Obolensk # 790039), *Bifidobacterium bifidum* 791 (Bioresource Center Russian National Collection of Industrial Microorganisms (BRCVKPM) # B-3300), *Bifidobacterium bifidum* 1 (SCPM-Obolensk # 900791), and *Bifidobacterium* (BRV 37 2000).

### 2.2. Cultivation of Strains

Lyophilized strains of *L. plantarum* 8 RA 3, *L. fermentum* 90 TC 4, and *L. fermentum* 39 were grown according to the method described previously [12], using liquid and agarized MRS nutrient medium (Syntex, Russia). Recovery and inoculation of strains of *B. bifidum* 1, *B. bifidum* 791, and *B. longum* 379 were carried out according to a similar scheme using the HMM liquid medium (hydrolyzate-milk medium, Syntex, Russia) and Bifidobacterium agar medium (HiMedia, India). We used cell suspensions of the third generation of each culture grown using the liquid media—MRS for lactic acid bacillus and HMM for bifidobacteria containing 10^8^ cells/ml. Each suspension was centrifuged for 10 min at 3000 g. To study antiviral activity, we used liquid media, supernatant fluid, and bacteria after centrifugation of 1 ml suspension.

### 2.3. Identification of Strains

Grown colonies of microorganisms were identified using the Bruker Daltonics Autoflex MALDI TOF mass spectrometer (Germany). The sample preparation was performed by direct application according to the standard protocol presented in the user manual. The mass spectra identification, recording, processing, and analysis were carried out using the BioTyper hardware-software complex.

### 2.4. Biochemical Property Study

The biochemical properties of the strains were confirmed using the BioMerieux API 50 CHL and API 20 A (France) test systems.

### 2.5. Whole Genome Sequencing

To conduct whole genome sequencing of the strains, genomic DNA was isolated using the QIAamp DNA Mini Kit QIAGEN commercial kit (Germany), and fragmentation was performed using the Applied Biosystems Covaris E210 ultrasonic fragmentation (USA) according to the manufacturer's instructions. The mixture purification and the 200-700 bp fragments selection were carried out using the Beckman Coulter Agencourt AMPure beads magnetic particles (USA) and New England Biolabs NEBNext Sizing Buffer (USA). Libraries were prepared using the Illumina Inc. TrueSeq kit (USA), and the sequencing was performed on the Illumina Inc. MiSeq platform (USA). The source reads were processed by Trimmomatic with standard parameters for Illumina. Then, the processed reads were used to assemble the de novo genome using the Spades, MIRA 4.0, and Newbler 2.6 programs.

### 2.6. Genome Annotation

Genome annotations were performed using the Prokka v. 1.11 utility [13] and the RAST genomic server (http://rast.nmpdr.org). The determinants of antibiotic resistance and pathogenicity were searched using software products available on the website of the Center for Genomic Epidemiology (http://www.cge.cbs.dtu.dk/): ResFinder 2.0, PathogenFinder, and PlasmidFinder programs [14–16].

### 2.7. The Determination of Probiotic Cytotoxicity for MDCK and VERO E6 Cells

The toxicity of all cell suspensions and the supernatant obtained from 1 ml of cell suspension, as well as microbiological media that were used to grow probiotic bacteria, were studied using the MTT test. Various bacterial concentrations were added, as well as the medium in which the bacteria were grown (supernatant from centrifugation of 1 ml of cell suspension) into 96-well plates (Greiner Bio-One) with MDCK cells (dog kidney cells). Cells were cultured for 72 hours, and cell viability was monitored by the MTT test (methylthiazolyldiphenyltetrazolium bromide) [17].

### 2.8. Antiviral Activity against Influenza A Virus Study

For antiviral activity analysis in vitro, the wells of a 96-well plate were inoculated with MDCK cell culture in MDCK DMEM (Gibco) medium supplemented with 5% bovine serum (Gibco) and antibiotics. The inoculating dose was 2 × 10^4^ cells per well. After the 90% monolayer formation (20 hours of incubation at 37°C in an atmosphere of 5% СО_2_), the cells were washed with serum-free medium. In a serological 96-well plate, twofold dilutions of the studied substances were prepared on a growth culture medium (DMEM Gibco, antibiotics, TRSC-trypsin 2 *μ*g/ml, 0.2% of bovine albumin) in a volume of 100 *μ*l. 100 *μ*l of the virus at a dose of 100 TCID_50_ was added to each well. The influenza A/Lipetsk/1V/2018 (H1N1 pdm09) (EPI_ISL_332798) and A/common gull/Saratov/1676/2018 (H5N6) (EPI_ISL_336925) virus strains were used in this work. The suspension was held for 60 min at a temperature of 37°C and introduced into the culture plate wells with MDCK cells. The MDCK cells were incubated at 37°C in an atmosphere of 5% СО_2_ for 72 h. Then, a neutral red was added to each well (final concentration -0.34%), cells were washed after 1.5 h, a dye extraction solution was added (0.1 M NH_4_H_2_PO_4_ and 96% ethanol in equal volumes), and the optical density of the released dye was determined on the BioRad 680 microplate reader at a wavelength of 490 nm using the Zemfira 2.0 software.

### 2.9. Antiviral Activity against SARS-CoV-2 Study

The antiviral activity was investigated against the SARS-CoV-2 virus of Australia/VIC01/2020 strain (GenBank: MT007544.1), kindly provided by the Western Pacific Office of the World Health Organization. A culture of Vero E6 cells (grass monkey kidney cells) was added to the wells of a 96-well plate in Needle MEM medium (Gibco) supplemented with 5% bovine serum (Gibco) and antibiotics. The inoculating dose is 1.5 × 10^4^ cells per well. After the 90–100% monolayer formation (48 hours of incubation at 37°C in an atmosphere of 5% СО_2_), the cells were washed with serum-free medium. The supernatant suspension or probiotic bacteria were diluted from 1 : 2 to 1 : 128 and introduced into the culture plate wells with Vero E6 cells. Cells were incubated for 2 h at 37°С and 5% СО_2_, after which 100 *μ*l of the virus was added at a dose of 0.1 TCID50/cell and incubated for 3 days at 37°С and 5% СО_2_. The results were determined using the MTT test, as described above, and the results were processed using the SOFTmaxPro 4.0 program (USA). The *χ*2 test was used to determine the statistical significance of differences in cell viability. The calculation was carried out using the statistical software package Statistica 6.0. A *p* value < 0.05 was considered significant.

## 3. Results

At the first stage of the work, phenotypic characteristics of strains were studied by studying their biochemical properties and protein mass spectra in order to select cultures intended for further studies of their entire genome. Then, whole genome sequencing of strains was performed, and the obtained nucleotide readings (reads) were used to assemble *de novo* genomes using modern bioinformatic programs. Genomes collected in the contigs format are registered in the GenBank/EMBL/DDBJ international database. The main genome characteristics of the studied strains are presented in Table 1.

### 3.1. Analysis of the Determinants of Pathogenicity and Antibiotic Resistance

According to the results, all investigated strains are not pathogenic for humans, and determinants of antibiotic resistance and integrated plasmids were not found in their genomes. Molecular efflux pumps of the MatE and MFS families were found in all *Lactobacillus*, which cause bacterial resistance to gentamicin, cefotaxime, tetracycline, ciprofloxacin, furazolidone, vancomycin, and sulfonamides (Table 1).

All *Bifidobacterium* have molecular efflux pumps of the MatE family, which cause the resistance of microorganisms to ampicillin, tetracycline, erythromycin, gentamicin, cefepime, cefotaxime, ciprofloxacin, furazolidone, polymyxin, and sulfamanidamicin. In addition, the cytoplasmic protein tetW was found in *B. longum* 379, which protects the ribosome from the effects of tetracycline (Table 1). No genes responsible for the synthesis of bacteriocins were identified.

### 3.2. Analysis of the Determinants of Sugar Metabolism

Genomic determinants of sugar metabolism were analyzed using the RAST server (http://www.nmpdr.org/). It was found that the subsystem of sugar metabolism in *L. plantarum* 8 RA 3 consists of 426 determinants. Due to the fact that this microorganism has a facultatively heteroenzymatic type of metabolism, its genome contains determinants of two pathways of sugar metabolism, fructose bisphosphate, and pentose phosphate, which allow the fermentation of sugars with the formation of acetic and lactic acids, formate, and ethanol. Determinants of the metabolism of monosaccharides were found in the genome: mannose, ribose, rhamnose, sorbose, arabinose, etc., di- and oligosaccharides, fructooligosaccharides and raffinose, amino sugars-chitin, N-acetylglucosamine, and glycogen (Table 1).

In the *L. fermentum* 90 TC 4 strain, this subsystem is represented by 113 determinants. The strain belongs to obligate heteroenzymatic group C lactobacilli; therefore, its genome lacks the main enzymes of the fructose bisphosphate pathway, aldolase, and triose phosphate-isomerase. However, the determinants of the pentose phosphate pathway are presented, which is the main variant of carbohydrate metabolism and allows fermentation of the sugar with the formation of lactic acid. The strain is distinguished by the “poor” spectrum of utilized sugars; it is unable to ferment mannose, ribose, and arabinose, which is due to the absence of individual genes (RbsD permease, phosphotransferase system (PTS ScrA), or whole operons (arabinose operon)) (Table 1).

The sugar metabolism subsystem of *L. fermentum 39* strain consists of 118 determinants. This strain also belongs to the group of heterofermentative lactobacilli of group C. Aldolase and triose phosphate-isomerase are absent in the genome, but the determinants of the pentose phosphate pathway are presented, which allows the fermentation of sugars to form lactic, acetic acids, and CO_2_. In the genome, the operons of metabolism and transport of mono- and disaccharides, riboflavin, and glycerol, which are absent in *L. fermentum* 90 TC 4, are determined (Table 1).

It has been established that the fructose-6-phosphate phosphoketolase pathway is the main pathway of carbohydrate metabolism in all studied *Bifidobacterium*. The products are lactic acid, acetic acid, and ethanol. The subsystems of sugar metabolism in *B. longum* 379, *B. bifidum* 1, and *B. bifidum* 791 consist of 199, 175, and 207 determinants, respectively. The genome of *B. longum* 379 contains determinants responsible for the utilization of monosaccharides (xylose, ribose, arabinose), disaccharides (sucrose, maltose, lactose, raffinose, phosphooligosaccharides), amino sugars, and glycogen. *B. bifidum* 1 and *B. bifidum* 791 have a low ability to metabolize monosaccharides; however, they are active against di-, oligosaccharides, and amino sugars-chitin, and N-acetylglucosamine. In addition, *B. bifidum* 791 is able to metabolize glycogen (Table 1).

### 3.3. Determinants of Exopolysaccharide Synthesis

It was found that the genome of all *Lactobacillus* contains the Eps operon responsible for the synthesis of exopolysaccharides, consisting of the enzymes EpsB (magnesium-dependent protein tyrosine phosphatase), EpsC (transmembrane tyrosine kinase), EpsD (tyrosine kinase), EpsE (galactophosphate), and polysaccharide transferase (Table 1). Analysis of this cluster suggested that all studied bacteria are capable of producing heteropolysaccharides consisting of a repeating unit of glucose and galactose.

Determinants of exopolysaccharide synthesis were found in the genome of *B. longum* strain 379: genes for rhamnose synthesis and genes responsible for the formation of sortase-dependent pili and cell wall lipoproteins. In the *B. bifidum* 1 strain, genes for the synthesis of exopolysaccharides were not found; however, there are determinants responsible for the formation of sortase-dependent pili and lipoproteins. The genome of *B. bifidum* 791 also contains genes for the synthesis of exopolysaccharides, including genes for the synthesis of rhamnose and genes of sortase-dependent pili and cell wall lipoproteins.

Thus, it was found that the studied bacteria are nonpathogenic for humans and do not contain transmissible-type antibiotic resistance genes and integrated plasmids. Resistance to antibiotics of various classes is explained by the presence of molecular efflux pumps of the MatE family and the protective ribosomal protein TetW. The studied *Lactobacillus* is capable of synthesizing acetic and lactic acid, formate, ethanol, one of the types of bacteriocins from the plantaricin group (L. plantarum 8 RA 3), and extracellular polysaccharides. All *Bifidobacterium* are able to utilize amino sugar and glycogen, di- and oligosaccharides, and acetic and lactic acids. Determinants responsible for the formation of sortase-dependent pili and cell wall lipoproteins were found in the genomes of all *Bifidobacterium*. *B. longum 379*, in contrast to *B. bifidum 791* and *B. bifidum 1*, is able to utilize monosaccharides (xylose, ribose, arabinose). *B. longum 379* and *B. bifidum 791* contain determinants of exopolysaccharide synthesis, which are absent in *B. bifidum 1*.

### 3.4. The Toxicity Bacteria Suspensions and Culture Fluid

**Figure 1:**
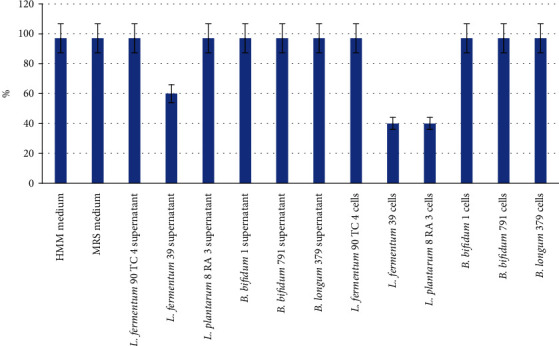
The number (in %) of viable MDCK cells after culturing for 72 hours with investigated components of probiotic bacteria liquid cultures. The results are average values ± standard deviations from three independent experiments. *p* < 0.05.

The toxicity of all bacteria suspensions and the supernatant obtained from 1 ml of bacteria suspension, as well as microbiological media that were used to grow probiotic bacteria, were studied in the MTT test. The results are presented in Figure 1.

As can be seen, the *L. fermentum* 39 and *L. plantarum* 8 RA 3 cell suspensions, as well as the supernatant obtained from the *L. fermentum* 39 cell suspension centrifugation, were toxic to MDCK cells. The remaining components studied in this work were nontoxic to mammalian cells.

### 3.5. The Antiviral Activity against Influenza A Virus Study

**Figure 2:**
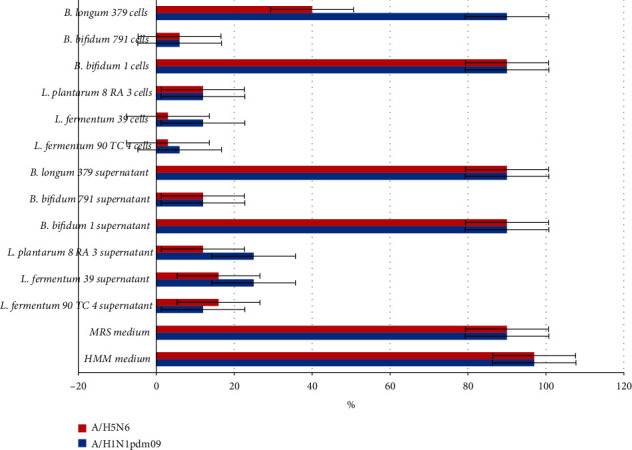
The smallest amount (in % of the initial suspension of bacteria or culture medium) that protects 50% of MDCK cells after infection with influenza viruses. Red color indicates the A/Lipetsk/1V/2018 (H1N1 pdm09) virus infection; blue color indicates the A/common gull/Saratov/1676/2018 (H5N6) virus infection. The results are average values ± standard deviations from three independent experiments. *p* < 0.05.

The antiviral activity study results are presented in Figure 2.

As can be seen from the data in Figure 2, the HMM medium did not have antiviral activity, in contrast to the MRS medium, which moderately inhibited the influenza virus reproduction. The supernatant obtained by centrifuging 1 ml of *L. fermentum* 90 TC 4 culture and the supernatant of *B. bifidum* 791 culture showed pronounced activity against the epidemic influenza A/Lipetsk/1V/2018 virus (H1N1 pdm09). They were active up until a dilution to 12.5% of the initial suspension. The cells of these bacterial cultures suppressed the influenza virus epidemic strain reproduction before diluting the suspension of bacterial cells to 6.25% of the initial suspension. The L*. plantarum* 8 RA 3 bacteria reduced the virus reproduction by 50% upon cell suspension dilution to 12.5% of the initial suspension. Supernatants and *B. bifidum* 1 and *B. longum* 379 cells showed a decrease in the virus cytopathic effect on MDCK cells at the same level as the medium in which these bacteria were grown.

The supernatants of *L. plantarum* 8 RA 3 and *B. bifidum* 791 bacterial cultures showed activity against the highly pathogenic avian influenza A/chicken/Nghe An/27VTC/2018 (H5N6) strain until dilution to 12.5% of the initial concentration. The virus reproduction was reduced by 50% with *L. fermentum* 90 TC 4 and *L. fermentum* 39 cell suspension at a concentration of 3.13% of the initial suspension and *B. bifidum* 791 cell suspension at a concentration of 6.25% of the initial one.

**Figure 3:**
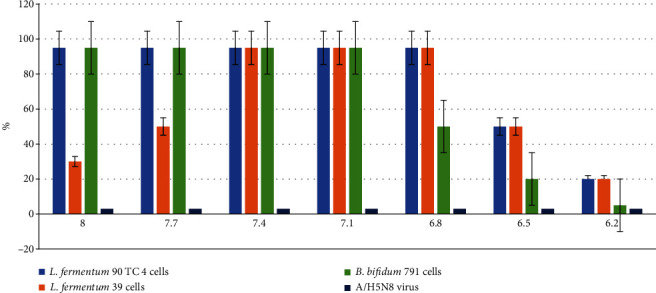
The number (in %) of viable MDCK cells after virus infection at a dose of 100 TCID/_50_ and during incubation with different concentrations of bacteria. Along the abscissa axis is the logarithm of bacteria concentration, and along the ordinate axis is the % of viable cells. A positive control (uninfected cells were cultured without probiotic bacteria) is 100% viable cells. The results are average values ± standard deviations from three independent experiments. *p* < 0.05.

Thus, the *Lactobacillus fermentum* bacteria of 90 TC 4 strain, *Lactobacillus fermentum* of 39 strain, and *Bifidobacterium bifidum* of 791 strain appeared to be the most promising cultures for further antiviral activity studies. They were further investigated. The results are presented in Figure 3.

As can be seen in Figure 3, all three cultures of probiotic bacteria showed pronounced antiviral activity against the highly pathogenic strain of the influenza A/common gull/Saratov/1676/2018 (H5N6) virus to a concentration of 10^7.1^, and the *Lactobacillus fermentum* of 90 TC 4 strain and *Lactobacillus fermentum* of 39 strain bacterial suspension to a concentration of 10^6.8^ completely suppressed the multiplication of influenza virus in MDCK cells.

### 3.6. The Antiviral Activity against SARS-CoV-2 Study

**Figure 4:**
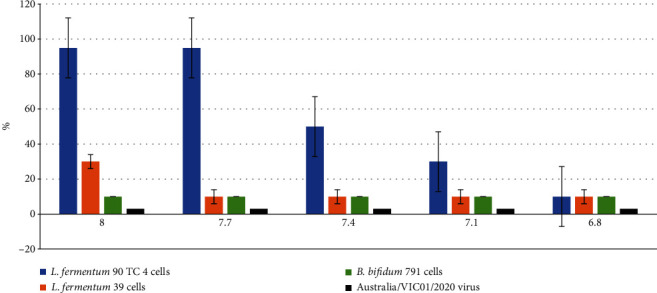
The number (in %) of viable VERO E6 cells after virus infection at a dose of 100 TCID/_50_ and during incubation with different concentrations of bacteria. Along the abscissa axis is the logarithm of bacteria concentration, and along the ordinate axis is the % of viable cells. A positive control (uninfected cells were cultured without probiotic bacteria) is 100% viable cells. The results are average values ± standard deviations from three independent experiments. *p* < 0.05.

The antiviral activity against SARS-CoV-2 was studied on VERO E6 cells according to a scheme, described in Materials and Methods. The results are presented in Figure 4.

As can be seen from the data in Figure 4, only *L. fermentum* 90 TC 4 cells produced an antiviral effect against the SARS-CoV-2 virus. Nevertheless, in our opinion, these are encouraging results that allow us to recommend the probiotic bacteria for COVID-19 prevention.

## 4. Discussion

During the influenza pandemic and sometimes during seasonal epidemics, gastrointestinal manifestations of the disease are observed, especially in children. In addition, it has been shown that the highly pathogenic avian influenza virus often infects the intestinal epithelium during human infection [10].

The situation associated with the incidence of zoonotic influenza remains tense. More than 860 cases of human infection with highly pathogenic avian influenza A/H5N1 virus, with an increased incidence of children under 15 years and a mortality rate of over 50%, have been registered in the world [18]. Since 2013, the carryover of A/H7N9 influenza virus from birds to humans has been confirmed in more than 1,500 cases, resulting in severe pneumonia with multiple organ failures and a mortality rate of approximately 40% (data as of January 31, 2021) [19]. If these viruses begin to be effectively carried over from person to person, they can cause an influenza pandemic with very drastic consequences.

In December 2019, an outbreak of disease caused by the SARS-CoV-2 coronavirus was recorded in Wuhan, China. In March 11, 2020, the WHO President announced the pandemic breakout [20]. As for all respiratory viruses, the site of SARS-CoV-2 entry is the upper respiratory tract epithelial cells, but it has been shown that the new coronavirus often infects the intestinal tract epithelium [11]. The angiotensin converting enzyme 2 (ACE2), which is a receptor for the SARS-CoV-2 virus [21], is presented in abundance not only in the alveolar epithelial cells of the lungs but also in enterocytes of the small intestine, in the upper esophagus, liver, and colon [22]. Moreover, it was indicated that viral RNA excretion in feces was detected longer than in swabs from the nasopharynx [23].

At the present stage, a new direction in the treatment and prevention of viral infections is associated with the use of probiotic bacteria strains of *Lactobacillus* and *Bifidobacterium* genera. Research and clinical trials are currently being conducted in the world that are devoted to them [24]. It has been shown that probiotic bacteria are capable of producing an antiviral effect by stimulating the general immune response. They increase the induction of interleukins and activation of macrophages, natural killer cells, and T-helpers and stimulate the production of immunoglobulins, in particular IgA, type I interferons [25, 26]. Several investigators considered the ability of bacteria to adhere to the virus particles as a mechanism of probiotic antiviral action. It was found that individual strains of *Lactobacillus* are able to adsorb the virus of vesicular stomatitis particles (*Rabdoviridae* family), А and В4 Coxsackie virus (*Picornaviridae* family), A71 enterovirus (*Picornaviridae* family), and human noroviruses (*Caliciviridae* family) [25–29]. The mechanism of bacterial cell and viral particle interaction is interpreted as physical adsorption by most investigators, which is carried out due to nonspecific Van der Waals forces and is strain-specific [28, 29].

Active metabolites produced by probiotic microorganisms, such as lactate, hydrogen peroxide, short-chain fatty acids, and bacteriocins, have a direct virucidal effect, as they cause a decrease in the medium pH, a violation of the viral particle adhesion mechanism due to changes in their receptor properties [28, 30–32]. All too often, the whole complex of extracellular substances has an antiviral effect, and it is not possible to isolate a separate active substance [33]. There is also evidence that lactic acid bacilli exopolysaccharides, which are homo- or heteropolysaccharides of various chemical compositions and also having strain specificity, have a pronounced antiviral effect [34].

All probiotic strains of *Lactobacillus* genus in this study have a range of properties that can provide an antiviral effect—the production of organic acids, ethanol, and exopolysaccharides. The *L. plantarum* 8 RA 3 strain was considered promising in this regard, since it is the most metabolically active, capable of synthesizing plantaricin, and has high antagonistic activity against pathogenic and opportunistic pathogenic bacteria. However, this strain did not show high antiviral activity in the experiment with influenza A viruses, in contrast to *L. fermentum* 90 TC 4 and *L. fermentum* 39 bacteria. In addition, culture fluids (supernatants) containing only bacterial metabolites of *L. fermentum* 90 TC 4 and *L. fermentum* 39 also exhibited antiviral activity, and *L. fermentum* 90 TC 4 bacteria showed antiviral activity in the experiment with SARS-CoV-2.

All the studied cultures are capable of producing heteropolysaccharides, consisting of galactose and glucose residues; however, *L. plantarum* 8 RA 3 polysaccharides differ in spatial conformation and other characteristics from exopolysaccharides (EPS) of *L. fermentum* 90 TC 4 and *L. fermentum* 39. Perhaps, this is one of the factors causing differences in their antiviral activity, which is consistent with the data of Kim et al. [34].

As for bacteria of *Bifidobacterium* genus, the scientific literature describes the assumptive mechanisms of their antiviral action—a stimulation of the macroorganism immune response, a production of organic acids, ethanol, exopolysaccharides, and also cell wall secreted lipoproteins (LpAs), which are capable of obstructing the interaction of viral particles with human mucous membrane receptors and thereby preventing the infection progression [24].

According to present knowledge, the representatives of *B. adolescentis*, *B. longum*, and *B. breve* species show activity against viruses, and it has been shown that this is a strain-specific feature [24, 26, 32]. According to data obtained from experimental studies conducted in France, Korea, and Colombia in 2015-2017, the representatives of *B. longum* species showed pronounced antiviral activity against human entero- and rotavirus [24, 26, 32].

However, in our study, the *B. longum* 379 strain, despite being characterized as metabolically active, did not show activity against influenza A viruses—the epidemic A/Lipetsk/1V/2018 (H1N1) influenza virus and the highly pathogenic avian A/common gull/Saratov 1676/2018 (H5N6) influenza virus. Within the framework of this study, the antiviral activity of representatives of *B. bifidum* species, *B. bifidum* 1, and *B. bifidum* 791 strains was first studied. These cultures do not utilize the monosaccharides; that is, they have a less pronounced metabolic potential, and *B. bifidum* 1 strain is not capable of synthesizing exopolysaccharides, which probably causes absence of its antiviral activity.

Only the *B. bifidum* 791 strain showed a pronounced activity against influenza A viruses; moreover, the culture fluid has also a virucidal effect. This may be due to strain-specific features—structural features of the cell wall, as well as the ability to produce secreted (free) lipoproteins to the culture medium.

Thus, our data showing that probiotic bacteria inhibit the highly pathogenic respiratory virus reproduction in cell culture is important for the prevention and treatment of diseases caused by respiratory viruses that can affect the human intestinal tract. The acute relevance of this topic due to the epidemiological situation in the world makes it imperative to continue these studies.

There is a limitation of this study. Testing of antiviral activity was carried out *in vitro* in Madin-Darby canine kidney cells (MDCK) and green monkey kidney cells (Vero) due to their most sensitivity to influenza virus and SARS-CoV-2 virus, respectively [35, 36]. We plan to conduct the study in animals to confirm our conclusions on the viability to use probiotics when treating respiratory viral infections. After that, we plan to lead the clinical trial. Thus, the study by Wang et al. [37] showed that probiotics are promising for treating viral pneumonia; their application decreases inflammatory reactions, induces both inherent and adaptive immune responses, and prevents consecutive bacterial infections. As bacteria demonstrated in this study are already actively used as probiotics in Russia, we plan to lead the clinical trial in hospitals where probiotics will be included in the regular treatment of patients infected with respiratory viruses including SARS-CoV-2.

## Figures and Tables

**Table 1:** The main genome characteristics of the studied probiotic bacteria.

Bacteria	Main characteristics of the genome	Determinants of bacteriocin synthesis	Determinants of antibiotic resistance	Determinants of sugar metabolism	Determinants of exopolysaccharide synthesis
*L. plantarum* 8 RA 3	GenBank number LBDF00000000 Genome type draft Number of contigs 18 Genome size 3,330,093 bp Average coverage 250.0 GC composition 44.4% CDS quantity 2982	KLD40925.1, KLD40929.1, KLD40930.1, KLD40931.1, KLD40932.1, KLD40933.1 Contig 4 LBDF01000008.1	Efflux pumps *MatE* KLD43042.1 *MFS* KLD43077.1	426 determinants Fructose bisphosphate pathway Pentose phosphate pathway Utilization of fructooligosaccharides and raffinose: *Msm* KLD40778.1, *PTS* KLD43366.1, KLD43367.1, KLD43368.1, *SacA* KLD43120.1, *DexB* KLD43365.1, *Aga* KLD42863.1, *Man* KLD43356.1 и *BG* KLD41117.1 Utilization of amino sugars: *NagA* KLD43035.1, *NagB* KLD43152.1, *PTS NagT* KLD42190.1, *NagR* KLD40894.1, *Cbp* KLD42577.1 *ChiA* KLD43348.1 Glycogen metabolism: *GAT* KLD43421.1, *GS* KLD43423.1, *GP* KLD43424.1	*Eps operon* *EpsB KLD41507.1* *EpsC KLD40740.1* *EpsD KLD40739.1* *EpsE KLD41508.1* *EpsF KLD41509.1* *Contig 15* *LBDF01000011.1*

*L. fermentum* 90 TC-4	GenBank number LBDH00000000 Genome type draft Number of contigs 93 Genome size 1,822,484 bp Average coverage 250.0 GC composition 51.9% CDS 1620 quantity		Efflux pumps *MatE* KLD54124.1 *MFS* KLD53990.1	113 determinants Pentose phosphate Pathway utilization of disaccharides: *GalK* KLD53103.1, *FruK* KLD55067.1, *α-Gal* KLD53100.1, *β-Gal* WP_046949162.1, *mapA* P_046948545.1, *β*-glucosidase KLD43584.1	*Eps operon* *EpsB KLD49590.1* *EpsC KLD49588.1* *EpsD KLD49589.1* *Contig 15* *LBDH01000061.1*

*L. fermentum* 39	GenBank number LBDG00000000 Genome type draft Number of contigs 55 Genome size 1,829,655 bp Average coverage 250.0 GC composition 51.6% CDS quantity 1683		Efflux pump *MatE* KLD56150.1	118 determinants Pentose phosphate metabolic pathway Utilization of arabinose: AraR KLD56054.1, AraK KLD56053.1, AraA KLD56052.1, AraD KLD56052.1, AraT KLD56053.1 Glycerol utilization: GlpT KLD54015.1, GlpF KLD56071.1, GlpK KLD55877.1, GPD KLD55407.1 Riboflavin metabolism: RybD KLD54636.1, RSA KLD54605.1	*Eps operon* *EpsB KLD51897.1* *EpsC KLD51899.1* *EpsD KLD51898.1* *EpsE KLD51903.1* *Contig 20* *LBDG01000035.1*

*B. longum 379*	GenBank number LKUQ00000000 Genome type draft Number of contigs 24 Genome size 2,387,620 bp Average coverage 150.0 GC composition 60.2% CDS 1903 quantity		Efflux pump *MatE* KYJ82530.1 цитоплазматический белок *tetW* KYJ81078.1	199 determinants Fructose-6-phosphate phosphoketolase pathway Amino sugar metabolism: NagK KYJ78240.1, NagA KYJ78237.1, NagB KYJ78238.1, NagT KYJ81992.1, NagR KYJ82080.1 Glycogen metabolism: GAT KYJ81114.1, GS KYJ81082.1, GBr KYJ82453.1, GP KYJ83271.1, GdBr KYJ82081.1, AMse KYJ82084.1, MalE KYJ78457.1 Utilization of raffinose and fructooligosaccharides: MsmR KYJ82093.1, MsmE KYJ82142.1, MsmF KYJ82092.1, MsmG, KYJ82141.1, SacA KYJ78008.1, Aga KYJ83465.1	Genes for rhamnose synthesis, contig 5 LKUQ01000020.1 Capsular polysaccharide genes: Wzb KYJ83195.1, Wzc KYJ83223.1 Sortase dependent pili: SrtA KYJ83477, AP KYJ83476.1 Lipoproteins: Lgt KYJ83617.1, LspA KYJ77995.1

*B. bifidum 1*	GenBank NDXI00000000 Genome type draft Number of contigs 13 Genome size 2,198,027 bp Average coverage 385.0 GC composition 62.7% CDS 1521 quantity		Efflux pump *MatE* PDH98462.1	175 determinants Fructose-6-phosphate phosphoketolase pathway Amino sugar metabolism: NagK PDH98478.1, NagA PDH97531.1, NagB PDH98129.1, NagT PDH97280.1, CbsA PDH98222.1, Aga PDH97428.1	Sortase dependent pili: SrtA PDH97100.1, PDH97310.1 Lipoproteins: Lgt PDH98440.1, LspA PDH98074.1

*B. bifidum 791*	GenBank number LKUR00000000 Genome type draft Number of contigs 33 Genome size 2,285,457 bp Average coverage 150.0 GC composition 62.4% CDS 1769 quantity		Efflux pumps *MatE* KYJ84349.1, KYJ84414.1	207 determinants Fructose-6-phosphate phosphoketolase pathway Amino sugar metabolism: NagK KYJ84330, NagA KYJ85076.1, NagB KYJ85077.1, NagT KYJ85215.1, KYJ85216.1, NagR KYJ85078.1, CbsA KYJ83728.1, Aga KYJ84485.1 Glycogen metabolism: GAT KYJ83153.1, GS KYJ84446.1, GBr KYJ84933.1, GP KYJ84691.1, GdBr KYJ85154.1, AMse KYJ84544.1	*Genes for the synthesis of rhamnose*, *contig 3 LKUR01000023.1* Sortase dependent pili: SrtA KYJ84870.1, AP KYJ84871.1 Lipoproteins: Lgt *KYJ84380.1*, *LspA KYJ85145.1*

## Data Availability

Data are available on demand.

## References

[1] Arena M. P., Capozzi V., Spano G., Fiocco D. (2017). The potential of lactic acid bacteria to colonize biotic and abiotic surfaces and the investigation of their interactions and mechanisms. *Applied Microbiology and Biotechnology*.

[2] Abdelazez A., Abdelmotaal H., Zhu Z. T. (2018). Potential benefits of Lactobacillus plantarum as probiotic and its advantages in human health and industrial applications: a review. *Advances in Environmental Biology*.

[3] Clancy R. (2003). Immunobiotics and the probiotic evolution. *FEMS Immunology and Medical Microbiology*.

[4] Ljungh A., Wanstrom T. (2009). *Lactobacillus, molecular biology. From genomics to probiotics*.

[5] Mayo B., Sinderen D. (2010). *Bifidobacteria: genomics and molecular aspects*.

[6] Henderson B., Nibali L. (2016). *The human microbiota and chronic disease: dysbiosis as a cause of human pathology*.

[7] Al Kassaa I., Hober D., Hamze M., Chihib N. E., Drider D. (2014). Antiviral potential of lactic acid bacteria and their bacteriocins. *Probiotics Antimicrob Proteins*.

[8] Wang Z., Chai W., Burwinkel M. (2013). Inhibitory influence of Enterococcus faecium on the propagation of swine influenza a virus in vitro. *PLoS One*.

[9] Ducatez M. F., Webster R. G., Webby R. J. (2008). Animal influenza epidemiology. *Vaccine*.

[10] Zhang H., Li H. B., Lyu J. R. (2020). Specific ACE2 expression in small intestinal enterocytes may cause gastrointestinal symptoms and injury after 2019-nCoV infection. *International Journal of Infectious Diseases*.

[11] Tochilina A. G., Belova I. V., Solovieva I. V., Gorlova I. S., Ivanova T. P., Zhirnov V. A. (2016). Characteristics of biological and MOLECULAR-GENETIC properties of Lactobacillus fermentum 90 TC-4 PROBIOTIC STRAIN. *Journal of Microbiology, Epidemiology and Immunobiology*.

[12] Belova I. V., Tochilina A. G., Solovyeva I. V. (2016). Lactobacillus fermentum 90 TC-4 taxonomic status confirmation using whole genome sequencing and MALDI TOF mass spectrum. *Russian Journal of Genetics*.

[13] Seemann T. (2014). Prokka: rapid prokaryotic genome annotation. *Bioinformatics*.

[14] Cosentino S., Voldby Larsen M., Møller Aarestrup F., Lund O. (2013). PathogenFinder - distinguishing friend from foe using bacterial whole genome sequence data. *PLoS One*.

[15] Grissa I., Vergnaud G., Pourcel C. (2007). CRISPRFinder: a web tool to identify clustered regularly interspaced short palindromic repeats. *Nucleic Acids Research*.

[16] Briner A. E., Barrangou R. (2014). Lactobacillus buchneri genotyping on the basis of clustered regularly interspaced short palindromic repeat (CRISPR) locus diversity. *Applied and Environmental Microbiology*.

[17] Mosmann T. (1983). Rapid colorimetric assay for cellular growth and survival: application to proliferation and cytotoxicity assays. *Journal of Immunological Methods*.

[18] April 2020, http://www.who.int/influenza/human_animal_interface/H5N1_cumulative_table_archives/en/

[19] April 2020, http://www.fao.org/ag/againfo/programmes/en/empres/H7N9/situation_update.html

[20] World Health Organization *WHO Director-General’s opening remarks at the media briefing on COVID-19 – 11 March*.

[21] Li G., He X., Zhang L. (2020). Assessing ACE2 expression patterns in lung tissues in the pathogenesis of COVID-19. *Journal of Autoimmunity*.

[22] Zhang W., du R. H., Li B. (2020). Molecular and serological investigation of 2019-nCoV infected patients: implication of multiple shedding routes. *Emerging Microbes & Infections*.

[23] Huang C., Wang Y., Li X. (2020). Clinical features of patients infected with 2019 novel coronavirus in Wuhan, China. *Lancet*.

[24] el Kfoury K. A., Romond M. B., Scuotto A. (2017). Bifidobacteria-derived lipoproteins inhibit infection with coxsackievirus B4 in vitro. *International Journal of Antimicrobial Agents*.

[25] Arena M. P., Capozzi V., Russo P., Drider D., Spano G., Fiocco D. (2018). Immunobiosis and probiosis: antimicrobial activity of lactic acid bacteria with a focus on their antiviral and antifungal properties. *Applied Microbiology and Biotechnology*.

[26] Kang J. Y., Lee D. K., Ha N. J., Shin H. S. (2015). Antiviral effects of Lactobacillus ruminis SPM0211 and Bifidobacterium longum SPM1205 and SPM1206 on rotavirus-infected Caco-2 cells and a neonatal mouse model. *Journal of Microbiology*.

[27] Ang L. Y. E., Too H. K. I., Tan E. L. (2016). Antiviral activity of Lactobacillus reuteri Protectis against Coxsackievirus A and Enterovirus 71 infection in human skeletal muscle and colon cell lines. *Virology Journal*.

[28] Botić T., Danø T., Weingartl H., Cencič A. (2007). A novel eukaryotic cell culture model to study antiviral activity of potential probiotic bacteria. *International Journal of Food Microbiology*.

[29] Lei S., Ramesh A., Twitchell E. (2016). High protective efficacy of probiotics and rice bran against human norovirus infection and diarrhea in gnotobiotic pigs. *Frontiers in Microbiology*.

[30] Sunmola A. A., Ogbole O. O., Faleye T. O. C., Adetoye A., Adeniji J. A., Ayeni F. A. (2019). Antiviral potentials of Lactobacillus plantarum, Lactobacillus amylovorus, and Enterococcus hirae against selected Enterovirus. *Folia Microbiologia (Praha)*.

[31] Salminen S., Bouley C., Boutron M. C. (1998). Functional food science and gastrointestinal physiology and function. *The British Journal of Nutrition*.

[32] Olaya Galán N. N., Ulloa Rubiano J. C., Velez Reyes F. A., Fernandez Duarte K. P., Salas Cárdenas S. P., Gutierrez Fernandez M. F. (2016). In vitro antiviral activity of Lactobacillus casei and Bifidobacterium adolescentis against rotavirus infection monitored by NSP4 protein production. *Journal of Applied Microbiology*.

[33] Lange-Starke A., Petereit A., Truyen U., Braun P. G., Fehlhaber K., Albert T. (2014). Antiviral potential of selected starter cultures, bacteriocins and D, L-lactic acid. *Food and Environmental Virology*.

[34] Kim K., Lee G., Thanh H. D. (2018). Exopolysaccharide from Lactobacillus plantarum LRCC5310 offers protection against rotavirus-induced diarrhea and regulates inflammatory response. *Journal of Dairy Science*.

[35] Hamamoto I., Takaku H., Tashiro M., Yamamoto N. (2013). High yield production of influenza virus in Madin Darby canine kidney (MDCK) cells with stable knockdown of IRF7. *PLoS One*.

[36] Rosa R. B., Dantas W. M., do Nascimento J. C. F., da Silva M. V., de Oliveira R. N., Pena L. J. (2021). In vitro and in vivo models for studying SARS-CoV-2, the etiological agent responsible for COVID-19 pandemic. *Viruses*.

[37] Wang F., Pan B., Xu S. (2021). A meta-analysis reveals the effectiveness of probiotics and prebiotics against respiratory viral infection. *Bioscience Reports*.

